# Remission Trajectories During Low-Intensity Cognitive Behavioural Therapy for Obsessive–Compulsive Disorder: A 2-Year Naturalistic Follow-Up Study

**DOI:** 10.3390/medicina62101970

**Published:** 2026-10-12

**Authors:** Keiichiro Mukai, Naomi Matsuura, Yukihiko Hosoi, Masahiko Sakurai, Kyosuke Yamanishi, Hisato Matsunaga

**Affiliations:** 1 Department of Neuropsychiatry, Hyogo Medical University, 1-1 Mukogawa-cho, Nishinomiya 663-8501, Japank-yama@hyo-med.ac.jp (K.Y.);; 2 Graduate School of Education, Mie University, Kurimamachiya-cho, Tsu City 514-8507, Japan

**Keywords:** low-intensity cognitive behavioural therapy, obsessive-compulsive disorder, remission, long-term follow-up

## Abstract

*Background and Objectives*: Cognitive-behavioural therapy (CBT) for obsessive–compulsive disorder (OCD) has primarily been evaluated in short-term, high-intensity treatment protocols. However, remission trajectories during low-intensity CBT (LiCBT) delivered in routine clinical practice remain insufficiently characterised. The aim of this study was to examine 2-year remission trajectories among patients with OCD receiving LiCBT in routine outpatient care. *Materials and Methods*: This study was a secondary analysis of an ongoing prospective cohort of patients with OCD treated at Hyogo Medical University Hospital, Japan. Patients who had received inpatient treatment or high-intensity CBT were excluded. Symptom severity was assessed using the Yale–Brown Obsessive–Compulsive Scale at baseline, 1 year, and 2 years. Patients were retrospectively classified into early remission, delayed remission, and non-remission groups according to remission timing. *Results*: Of 62 eligible patients, 47 completed the 2-year follow-up and were included in the remission trajectory analyses. Remission was achieved in 18 patients at 1 year and in an additional 10 patients at 2 years. At baseline, Y-BOCS scores were significantly lower in the early remission group than in the non-remission group, whereas by the 1-year follow-up, Y-BOCS scores differed significantly across all three groups. The delayed remission group did not meet the remission criteria at 1 year and subsequently achieved remission by 2 years. *Conclusions*: In this naturalistic multimodal treatment setting that included LiCBT, remission followed heterogeneous temporal trajectories rather than a single uniform course. The findings suggest that remission should not be evaluated solely within the first year of treatment and highlight the clinical importance of longitudinal assessment in routine outpatient care.

## 1. Introduction

Obsessive–compulsive disorder (OCD) is a mental disorder characterised by intrusive thoughts and repetitive behaviours, often accompanied by marked distress and functional impairment. OCD affects approximately 1–3% of the general population [1] and is widely recognised as a chronic condition that can substantially reduce quality of life (QOL) and social functioning [2]. First-line treatments for OCD include pharmacotherapy with selective serotonin reuptake inhibitors (SSRIs) and cognitive-behavioural therapy (CBT), particularly exposure and response prevention (ERP), which is a well-established evidence-based intervention [3].

Most evidence supporting CBT for OCD has been derived from short-term clinical trials conducted under relatively standardised treatment protocols. In general, CBT for OCD has been studied mainly as an acute-phase intervention delivered at fixed intervals over a limited period [4,5]. These studies have provided important evidence for treatment efficacy under controlled conditions. However, in routine clinical practice, treatment is often less standardised, and symptom improvement may not be fully captured within a short observation period [6]. Long-term follow-up studies of OCD have suggested that remission may increase gradually over time [7], with some patients achieving remission only after extended treatment. This pattern suggests that the treatment outcome in OCD cannot be understood only in terms of whether remission occurs, but also when it occurs. However, relatively few long-term studies have clearly described whether later remission occurred in the context of ongoing intervention, naturalistic improvement, or both. Therefore, the temporal course of remission under sustained therapeutic contact remains insufficiently characterised. This issue is particularly relevant in routine outpatient care, where treatment often continues over extended periods and does not necessarily follow a single fixed short-term protocol. In such settings, some patients may improve relatively early, whereas others may show clinically meaningful improvement only after longer-term treatment. Thus, evaluating remission only at a single endpoint may overlook important heterogeneity in treatment course.

Despite strong evidence supporting ERP, its implementation in routine clinical practice remains limited. Barriers such as limited availability of trained therapists, patient reluctance, and the perceived burden of exposure procedures may reduce the feasibility of delivering high-intensity CBT (HiCBT) in real-world settings [8,9]. Therefore, many patients receive modified or lower-intensity forms of CBT, which may differ substantially from protocols evaluated in clinical trials [10,11,12,13,14]. Low-intensity CBT (LiCBT), which is delivered less frequently and involves less therapist contact, may be provided over a longer period than conventional higher-intensity formats [12], providing a useful context in which to examine such variation. Although prolonged LiCBT has not been well characterised in the OCD literature [13,14], this format may allow gradual improvement to emerge in some patients under sustained therapeutic contact. In some healthcare systems, including Japan, CBT is often delivered at a relatively low frequency (e.g., approximately once per month) in routine outpatient care [15]. The effectiveness of such lower-frequency CBT has been reported in prior studies [13,14], suggesting that meaningful clinical improvement may occur even under less intensive CBT. Another important but underexplored issue is heterogeneity in treatment entry conditions. Baseline clinical status and early treatment responsiveness may influence not only overall treatment outcomes but also the timing of remission. In routine clinical care, treatment optimisation may require consideration not only of which intervention is provided, but also when and for whom it is most effective. However, few studies have examined how these factors relate to remission trajectories during long-term LiCBT.

The aim of the present study was to examine the 2-year clinical course and remission trajectories of patients with OCD who received LiCBT in routine outpatient care following an initial phase of pharmacological treatment. Particular attention was paid to remission trajectory over time, and the study explored whether distinct patterns of early remission, delayed remission, and non-remission could be identified.

## 2. Materials and Methods

### 2.1. Study Design and Setting

The present study was a secondary analysis of data from an ongoing prospective cohort at our university hospital, originally established to investigate long-term clinical outcomes. In a previous report from our group, long-term outcomes of patients with OCD treated in specialised clinical settings were examined, including 10-year remission rates and predictors of long-term prognosis in a Japanese sample [6]. In the current analysis, we focused on the first two years of follow-up and retrospectively identified a subgroup of patients according to predefined treatment-related criteria. No additional data collection was conducted for this analysis, and participation did not impose any further burden on patients. The cohort initially comprised 122 adult outpatients who met Diagnostic and Statistical Manual of Mental Disorders (DSM)-IV or DSM-5 criteria for OCD [16] and were consecutively treated at our university hospital between April 2013 and May 2024. During the earlier study period, OCD diagnosis was made according to DSM-IV criteria using the Structured Clinical Interview for DSM-IV Axis I Disorders, Clinician Edition [16]. After our routine clinical practice transitioned to DSM-5 in 2022, OCD was diagnosed according to DSM-5 criteria using the Japanese version of the Structured Clinical Interview for DSM-5 Disorders, Research Version (SCID-5-RV) [17,18]. The core diagnostic features of OCD, namely the presence of obsessions and/or compulsions causing clinically significant distress or impairment, were generally consistent between DSM-IV and DSM-5.

### 2.2. Participants

**Figure 1:**
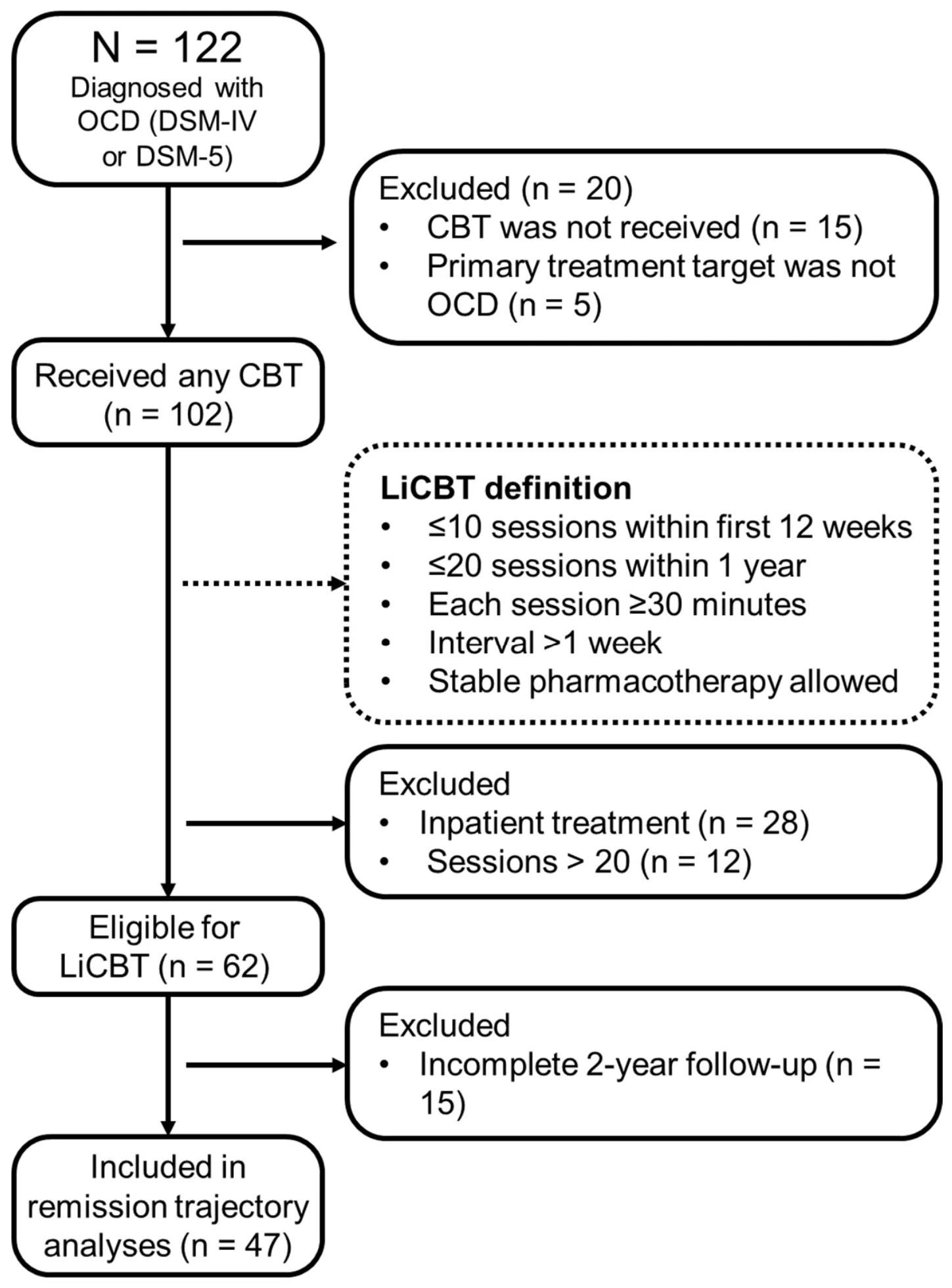
Flow of participant selection and application of LiCBT criteria.

The participants were adult patients diagnosed with OCD according to the DSM-IV or DSM-5 criteria and consecutively treated at the Department of Neuropsychiatry, Hyogo Medical University Hospital between April 2013 and May 2024. A total of 122 patients were initially identified from the clinical cohort. All patients received an explanation of the longitudinal prognosis study, including its purpose and procedures, and provided written informed consent to participate. Clinical data obtained as part of routine care before study enrollment were retrospectively incorporated into the longitudinal dataset in accordance with the approved study protocol. Patients were excluded if OCD was not the primary treatment target (Figure 1). Among the remaining patients, those who did not receive any form of CBT during the observation period were also excluded, in addition to patients who underwent inpatient treatment or HiCBT (defined as ≥21 sessions within the first year). LiCBT was operationally defined as lower-frequency ERP-based CBT delivered within routine outpatient care, generally involving ≤10 sessions within the first 12 weeks, ≤20 sessions within the first year and flexible intervals between sessions, with session durations of approximately 30 min and intervals typically longer than 1 week. The final remission trajectory analyses were restricted to patients who completed the 2-year follow-up.

The sample size was therefore determined by the number of patients who met the predefined eligibility criteria within the existing cohort during the study period, and no a priori sample size calculation was performed for this secondary analysis.

### 2.3. Psychometric Assessments

At baseline, demographic characteristics, family and medical history, clinical presentation, and course of illness were recorded. The principal obsessive–compulsive symptom dimension was assessed using the Japanese version of the Yale–Brown Obsessive–Compulsive Scale (Y-BOCS) Symptom Checklist [19,20,21]. The Y-BOCS is a clinician-rated instrument for evaluating the severity of OCD symptoms, with total scores ranging from 0 to 40. In clinical practice, symptom severity is commonly classified as subclinical (0–7), mild (8–15), moderate (16–23), severe (24–31), and extreme (32–40) [22]. In the present study, OCD severity was assessed at baseline and at 1- and 2-year follow-up points. Remission was defined as Y-BOCS total score below 8, consistent with prior studies [23]. Patients were retrospectively classified according to remission timing into early-remission, delayed-remission, and non-remission groups. The early-remission group comprised patients who achieved remission at the 1-year follow-up, whereas the delayed-remission group comprised patients who did not achieve remission at 1 year but achieved remission at the 2-year follow-up. The non-remission group comprised patients who did not achieve remission at either the 1- or 2-year follow-up.

Additional self-report measures included the State-Trait Anxiety Inventory (STAI) [24,25], which provides separate indices of state anxiety (STAI-S) and trait anxiety (STAI-T), Zung’s Self-Rating Depression Scale [26,27] and the World Health Organization Quality of Life Scale 26 for QOL [28]. Global functioning was evaluated with the Global Assessment of Functioning Scale (GAF), corresponding to Axis V of the DSM-IV [16]. These assessments were conducted at baseline and during follow-up.

### 2.4. Intervention

After the pretreatment assessments, each participant received pharmacotherapy with a standardised regimen of SSRIs, such as fluvoxamine (FLV) or paroxetine (PXT), a structured pharmacotherapy approach based on American Psychiatric Association treatment guidelines [29]. Pharmacotherapy was initiated at a daily dose of 50 mg of FLV or 10 mg of PXT. These agents were selected because they are among the SSRIs approved for OCD in Japan. If tolerated, the dosage was gradually increased over approximately 4 weeks to a maximum of 250 mg/day for FLV and 50 mg/day for PXT. In cases of insufficient treatment response, treatment strategies were adjusted according to prior treatment history. These adjustments included switching to alternative SSRIs, such as escitalopram or sertraline (maximum doses of 20 and 100 mg/day, respectively), or augmentation with antipsychotic agents such as aripiprazole or risperidone. All pharmacological decisions were made at the discretion of the treating psychiatrist as part of routine clinical care.

**Figure 2:**
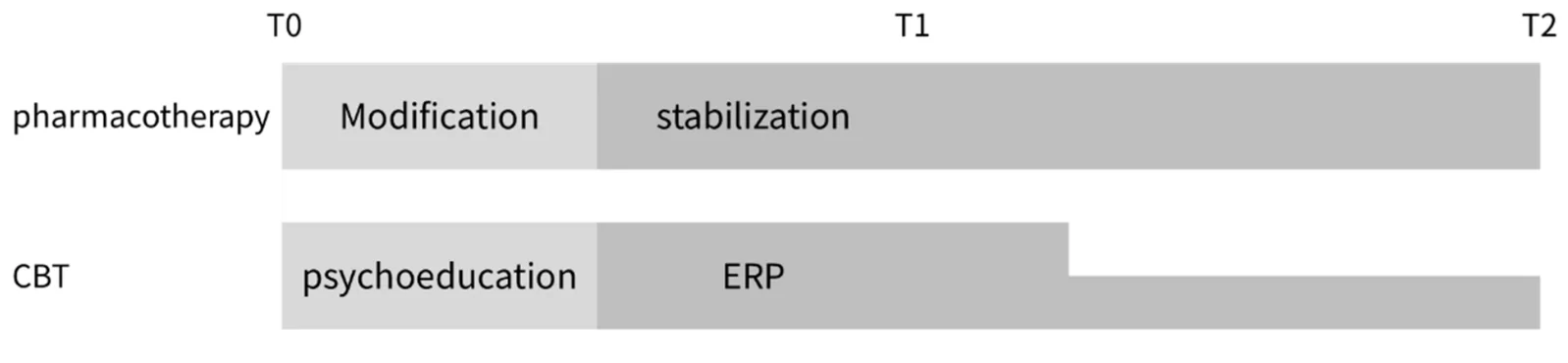
Stepwise treatment schedule integrating pharmacotherapy and CBT. Pharmacotherapy was adjusted from the initial visit based on patients’ treatment history and clinical status, followed by a stabilization phase. CBT was initiated with psychoeducation, and exposure and response prevention (ERP) was introduced after pharmacotherapy had been stabilized. Black segments indicate periods of treatment adjustment prior to stabilization.

Following the initial treatment phase, patients were managed with LiCBT as part of routine follow-up (Figure 2). This treatment approach was based on the procedure described in our previous study [15,30]. CBT, including ERP, was administered by treating psychiatrists, with each session lasting approximately 30 min. The treating psychiatrists had either completed formal CBT training or had at least 5 years of clinical experience in treating OCD, including experience under the supervision of an OCD specialist (H. M.). CBT was generally conducted using the treatment manual for OCD issued by the Japanese Society of Anxiety and Related Disorders [30]. CBT was initiated with a psychoeducation phase early in treatment, during which ERP was not yet implemented. This initial phase was primarily aimed at establishing a therapeutic alliance and clarifying target symptoms while adjusting and stabilising pharmacotherapy. ERP was introduced subsequently, generally after several weeks to months of pharmacological stabilisation. This stepwise approach reflects routine clinical practice, in which pharmacological stabilisation often precedes full implementation of ERP. The frequency and duration of visits were flexibly determined through shared decision-making between the patient and the treating psychiatrist, taking into account clinical status, patient preference, and practical considerations. CBT was delivered at each outpatient visit; therefore, the number of outpatient visits corresponded to the number of CBT sessions. CBT was continued during the second year as long as clinically significant OCD symptoms persisted.

### 2.5. Statistical Analysis

Descriptive statistics were calculated for all variables and are presented as means and standard deviations for continuous variables and frequencies and proportions for categorical variables. Group differences in continuous variables were examined using one-way analysis of variance or the Kruskal–Wallis test, as appropriate. Group differences in categorical variables were examined using chi-square tests with Yates’ correction or Fisher’s exact test, as appropriate, when expected cell counts were ≤5. Post hoc pairwise comparisons were adjusted using the Bonferroni correction.

All statistical analyses were conducted using IBM SPSS Statistics (version 20.0; IBM Corp., Armonk, NY, USA).

### 2.6. Ethics

The longitudinal study protocol was approved by the Ethics Committee of Hyogo Medical University (Approval No. 2080; 18 December 2015). Written informed consent was obtained from all participants. The approved protocol included both prospective follow-up and retrospective use of clinical data collected before study enrollment. The present analysis used data collected within this approved longitudinal study.

## 3. Results

**Figure 3:**
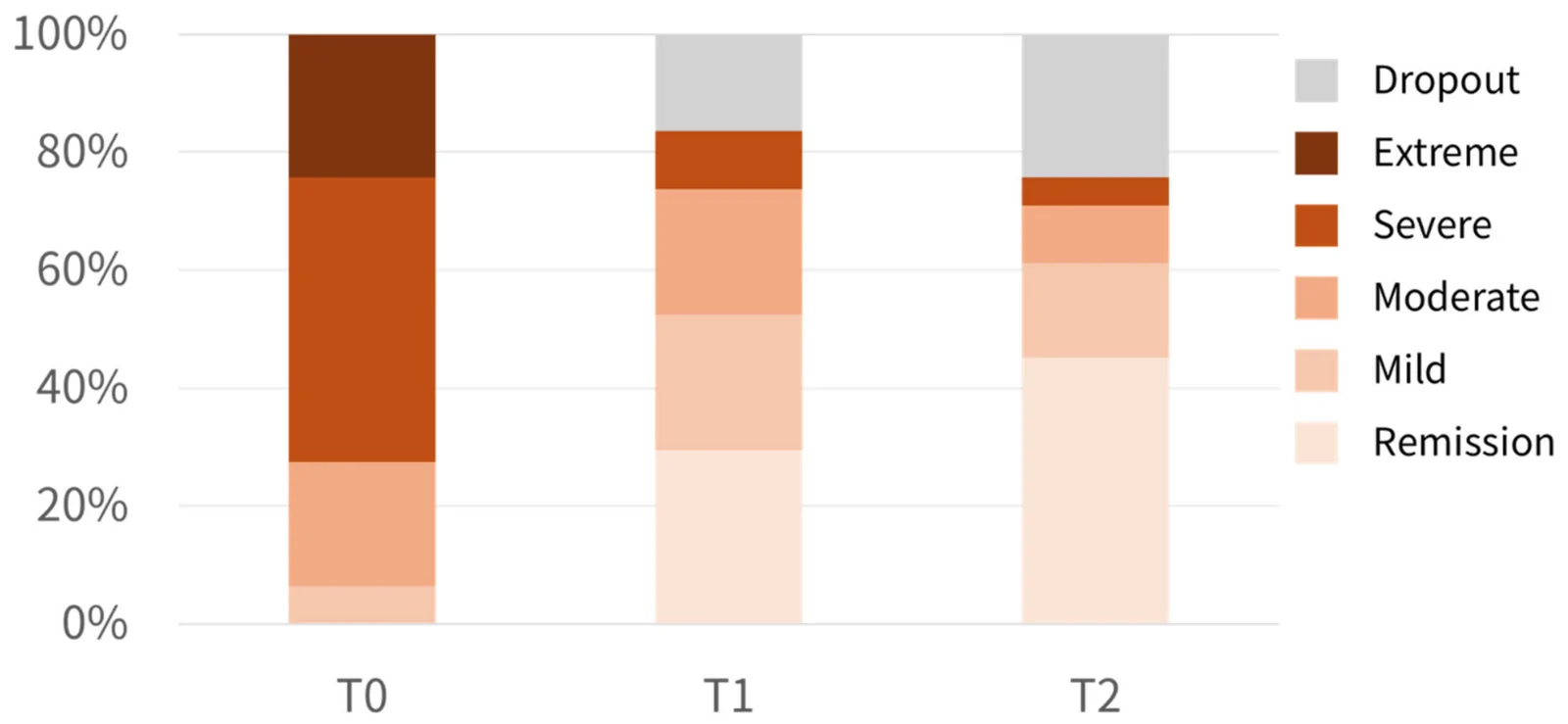
Distribution of Y-BOCS severity categories across assessment points. The stacked bars show the proportion of participants in each Yale–Brown Obsessive–Compulsive Scale (Y-BOCS) severity category at baseline (T0), 1-year follow-up (T1), and 2-year follow-up (T2). Severity categories were defined as remission (<8), mild (8–15), moderate (16–23), severe (24–31), and extreme (32–40).

A total of 62 patients met the eligibility criteria, of whom 47 completed the 2-year follow-up and were included in the remission trajectory analyses (Figure 1). Figure 3 illustrates the changes in OCD severity categories across the study period. At the 1-year follow-up, 18 patients achieved remission, and an additional 10 patients achieved remission between years 1 and 2, resulting in a total of 28 remitted patients at the 2-year follow-up. At baseline (T0), most patients were classified as severe (n = 30) or extreme (n = 15). At 1 year (T1), symptom severity shifted markedly toward lower categories, with no patients remaining in the extreme category and 18 patients achieving remission. This trend was maintained at 2 years (T2), with further reductions in the number of severe cases (n = 3).

Table 1 summarises the distribution of obsession and compulsion dimensions across the early remission, delayed remission, and non-remission groups. No significant between-group differences were observed in obsession dimensions after Bonferroni correction. Among compulsion dimensions, washing compulsions were significantly more common in the non-remission group (*p* = 0.002) and remained significant after Bonferroni correction.

No other symptom dimensions showed significant between-group differences. At baseline, significant group differences were observed in Y-BOCS total (*p* < 0.002) and GAF (*p* = 0.017) scores (Table 2). The early remission group had significantly lower Y-BOCS scores, and higher GAF scores, compared with the non-remission group.

**Figure 4:**
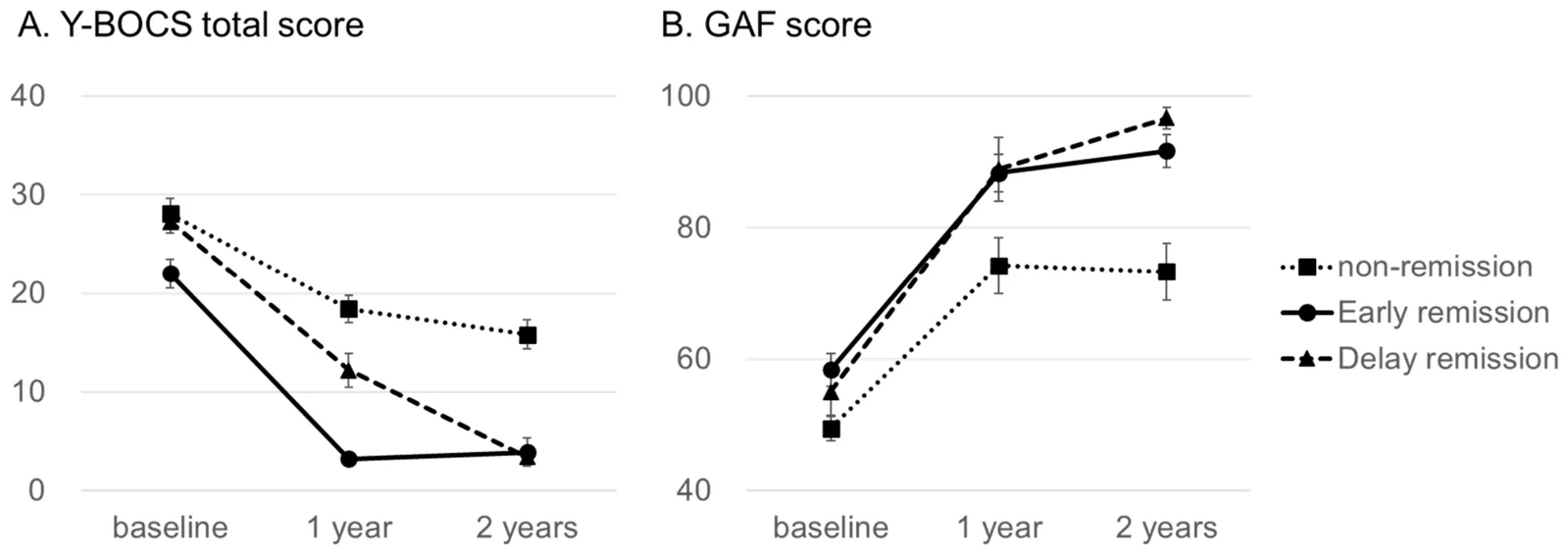
Changes in Y-BOCS total and GAF scores according to remission trajectory during the 2-year follow-up. (**A**) Mean Y-BOCS total scores and (**B**) mean GAF scores at baseline and at the 1- and 2-year follow-up assessments are shown for the early-remission, delayed-remission, and non-remission groups. Error bars represent standard errors of the mean (SE). Remission trajectories were defined according to the timing of remission based on Y-BOCS scores. Y-BOCS, Yale–Brown Obsessive–Compulsive Scale; GAF, Global Assessment of Functioning.

During the first year, treatment exposure was comparable across the three groups, with no significant differences in the number of outpatient visits, time to ERP initiation, or number of visits before ERP initiation (Table 3). Significant group differences were observed in Y-BOCS (*p* < 0.001), STAI-S (*p* = 0.035), and GAF (*p* = 0.014) scores, and in SSRI-equivalent dose (*p* = 0.005). Post hoc analyses showed that Y-BOCS scores differed significantly across all three groups, with the non-remission group having the highest scores, the early remission group the lowest scores, and the delayed remission group intermediate scores. The early remission group had lower SSRI-equivalent doses compared with the other groups. At 2 years, both remission groups showed significantly lower Y-BOCS (*p* < 0.001) and higher GAF (*p* < 0.001) scores compared with the non-remission group, with no significant differences between the early and delayed remission groups. Changes in Y-BOCS total and GAF scores across the 2-year follow-up period according to remission trajectory are shown in Figure 4. Changes in Y-BOCS total and GAF scores according to remission trajectory during the 2-year follow-up.

## 4. Discussion

The present study examined the 2-year clinical course of patients with OCD who received multimodal treatment including LiCBT in routine outpatient care and found that remission did not occur within a single fixed timeframe, such as 1 year. Rather, three remission trajectories—early remission, delayed remission, and non-remission—were descriptively identified over time. Consistent with their outcome-based definitions, the three groups showed different temporal patterns at each assessment point. These findings extend short-term treatment evidence by suggesting that clinically meaningful improvement may continue beyond the acute phase in a subset of patients. The present findings also highlight the heterogeneity of remission trajectories and the potential importance of evaluating treatment response longitudinally in routine clinical practice.

At the initial visit, 93.5% of patients were classified as having severe-to-moderate symptoms; this proportion decreased to 30.6% after 1 year and 17.7% after 2 years, indicating a gradual improvement in symptom severity over time. The remission rate at 1 year was 38.3%, and an additional 21.3% of patients achieved remission during the second year, resulting in a cumulative remission rate of 59.6% over 2 years. Previous long-term follow-up studies have reported that remission rates increase gradually over time, and the present findings are consistent with these observations [31,32,33]. The most comparable previous study is a 10-year follow-up study [6], which demonstrated a gradual increase in remission over time, with 56% of patients experiencing full remission during the follow-up period. However, unlike that study, the present sample excluded patients who received inpatient treatment or HiCBT. The relatively high remission rate in this study may partly reflect the inclusion of a relatively milder clinical population. Previous long-term studies have also suggested that early treatment response during the initial phase of treatment is associated with subsequent long-term outcomes, highlighting the clinical importance of improvement during the first year of treatment. Notably, our results further suggest that remission may continue beyond the first year under sustained therapeutic contact. In addition, a substantial proportion of patients achieve remission within the first 2 years of treatment [34]. Because the present study specifically focused on the initial 2-year treatment period, the findings may have clinically important implications regarding medium- to long-term outcomes in OCD. LiCBT was operationally defined as continuous ERP-based intervention delivered within routine outpatient care, allowing examination of both its potential clinical utility and its limitations. From a mechanistic perspective, HiCBT and LiCBT are not necessarily distinguished by fundamentally different psychological or neurobiological mechanisms, as both can incorporate ERP. Rather, LiCBT is primarily characterized by a lower dose of therapist contact and greater reliance on self-help or independently implemented therapeutic activities, whereas high-intensity approaches deliver a greater amount of therapist-supported ERP within a shorter period [35,36]. Thus, differences between these approaches may primarily reflect the intensity and mode of treatment delivery rather than distinct underlying therapeutic mechanisms. Taken together, these findings suggest the need for further studies comparing LiCBT with interventions of different treatment intensities and clarifying its role in real-world clinical implementation.

By the 1-year follow-up, Y-BOCS scores differed significantly across all three groups, and the trajectories clearly separated at this point. Given that the frequency of LiCBT sessions was generally comparable across groups, these findings indicate that factors other than CBT intensity may underlie the group differences. At the initial assessment, the early remission group showed significantly lower Y-BOCS scores compared with the non-remission group, whereas no significant difference was observed between the delayed remission and non-remission groups. From the perspective of stepped care, the National Institute for Health and Care Excellence guidelines recommend adjusting treatment intensity according to symptom severity and clinical needs [37]. In particular, lower-intensity CBT is recommended for patients with relatively milder symptoms. Consistent with this recommendation, the present findings suggest that lower symptom severity at the initial assessment was associated with early remission, supporting the clinical utility of actively considering LiCBT in selected patients within routine practice. This finding is consistent with previous studies and aligns with evidence that global functioning level along with symptom severity is associated with treatment outcome [7,38,39]. Furthermore, the SSRI doses at the 1-year follow-up were lower in our early remission group, although the clinical significance of this association remains unclear. Treatment during the first year included pharmacotherapy, psychoeducation, and ongoing ERP, which may have acted either independently or synergistically. In particular, pharmacotherapy administered prior to CBT may have been associated with greater readiness for treatment [40]. From a clinical perspective, it may also be important to consider the extent to which pharmacotherapy should be optimised before initiating CBT. In addition to pharmacotherapy, readiness for CBT may also have been associated with factors such as patient motivation and partial symptom improvement prior to the initiation of ERP-based intervention [41]. These findings highlight the importance of assessing both treatment responsiveness and readiness before initiating CBT, and of considering interventions tailored to these factors. Studies with larger samples are needed to examine these interrelated factors using more comprehensive statistical approaches.

Our delayed remission group showed relatively severe symptoms at baseline and did not achieve remission at 1 year but subsequently achieved by the 2-year follow-up. These findings suggest that a subgroup of patients achieve remission later during medium- to long-term multimodal treatment including continuous ERP. Although the early and delayed remission groups followed different temporal trajectories, their outcomes converged by 2 years. Most previous studies on CBT for OCD focused on short-term outcomes under acute treatment protocols, typically involving weekly sessions over 10–12 weeks. Therefore, treatment response has often been evaluated according to whether remission was achieved within a relatively fixed period. By contrast, the present findings suggest that remission during long-term multimodal treatment including LiCBT should not be evaluated solely within the first year. Even when remission was not achieved at 1 year, some patients continued to improve and subsequently reached remission during longer-term follow-up. Similar delayed improvement has been reported in studies examining booster CBT following standard acute-phase treatment [42]. These findings may reflect ongoing treatment responsiveness and may help identify patients who may continue to improve during ongoing ERP-based intervention. By contrast, the non-remission group continued to show higher symptom severity at the 2-year follow-up. These findings highlight the importance of longitudinally monitoring symptom trajectory during ongoing treatment. In patients showing limited continued improvement, more intensive ERP-based intervention may need to be considered.

Several limitations should be considered. First, this was a single-site observational study with a relatively small sample, and the subgroup analyses should be regarded as exploratory. Given the single-center design and the specific eligibility criteria applied in this secondary analysis, the findings may not be directly generalizable to the broader population of patients with OCD in Japan or other countries. Second, the absence of a control group limits causal interpretation of symptom change over time. In particular, the contributions of LiCBT, pharmacotherapy, and naturalistic improvement could not be disentangled. Third, the differences in SSRI-equivalent dose between the early and delayed remission groups should not be interpreted as evidence of a direct dose–response relationship. Further studies with larger samples and more structured assessment of treatment exposure are needed to clarify the factors associated with remission timing under routine care.

## 5. Conclusions

The present study suggests that remission during naturalistic multimodal treatment including LiCBT may follow multiple temporal patterns rather than a single uniform course. The early remission, delayed remission, and non-remission groups differed in the temporal pattern of remission. In particular, the convergence of the early and delayed remission groups by 2 years highlights the clinical importance of evaluating remission longitudinally. These findings suggest that, in routine practice, especially for CBT, understanding when remission occurs may be as important as determining whether it occurs.

## Figures and Tables

**Table 1:** Obsession/Compulsion Dimensions by Outcome Group.

n (%)	Early Remission		Delayed Remission		Non-Remission		*p*
	n = 18		n = 10		n = 19		
Obsession							
Aggression	15	(83.3)	6	(60.0)	10	(52.6)	0.133
Contamination	8	(44.4)	4	(40.0)	15	(78.9)	0.053
Sexual	1	(5.6)	—		—		0.596
Hoarding	0	(0.0)	2	(20.0)	—		0.042
Religious	1	(5.6)	—		—		0.596
Symmetry	—		—		—		—
Somatic	1	(5.6)	—		2	(10.5)	0.789
Other Obsession	1	(5.6)	1	(10.0)	1	(5.3)	1.000
Compulsion							
Washing	7	(38.9)	4	(40.0)	17	(89.5)	0.002
Checking	15	(83.3)	8	(80.0)	11	(57.9)	0.213
Repeating	2	(11.1)	1	(10.0)	2	(10.5)	1.000
Counting	—		—		—		—
Ordering	2	(11.1)	—		—		0.183
Hoarding	—		1	(10.0)	1	(5.3)	0.684
Other compulsions	—		—		—		—

Note. *p* values were calculated using Fisher’s exact test. Bonferroni correction was applied separately to obsession and compulsion dimensions. The corrected significance levels were *p* < 0.0071 (0.05/7) for obsession dimensions and *p* < 0.010 (0.05/5) for compulsion dimensions.

**Table 2:** Baseline Clinical and Demographic Characteristics.

	Early Remission		Delayed Remission		Non-Remission				
Variable	Mean (SD)/n (%)		Mean (SD)/n (%)		Mean (SD)/n (%)		F/H	*p*	Post Hoc
	n = 18		n = 10		n = 19				
Age	37.8	(10.0)	40.7	(13.2)	36.7	(11.4)	0.419	0.66	NS
Onset age	26.3	(8.9)	28.6	(16.2)	25.8	(11.3)	0.199	0.821	NS
Duration	11.5	(11.2)	11.9	(12.8)	10.9	(8.6)	0.033	0.968	NS
Education	14.5	(2.1)	13.4	(5.0)	14.8	(1.4)	0.859	0.43	NS
Sex (male)	8	(44.4)	4	(40.0)	4	(21.1)	—	0.323	NS
Married	3	(16.7)	2	(20.0)	4	(21.1)	—	1.000	NS
Employed	10	(55.6)	5	(50.0)	11	(57.9)	—	0.932	NS
Living status (with cohabitants)	15	(83.3)	6	(60.0)	14	(73.7)	—	0.397	NS
SSRI use	11	(61.1)	2	(20.0)	13	(68.4)	—	0.044	NS
SSRI dose (imipramine equivalents)	63.3	(74.1)	15.0	(31.6)	96.1	(83.2)	8.034	0.018	NR > DR *
Antipsychotic use	—		—		5	(26.3)	—	—	—
Antipsychotic dose (chlorpromazine equivalents)	0.0	(0.0)	0.0	(0.0)	26.3	(47.5)	8.041	0.018	NR > ER *
Y-BOCS	22.0	(6.3)	27.3	(3.7)	28.1	(6.5)	5.393	0.008	NR > ER **
SDS	49.6	(9.0)	49.1	(10.1)	55.0	(9.7)	1.923	0.158	NS
STAI-State	52.0	(13.5)	52.1	(11.8)	58.3	(13.6)	1.254	0.295	NS
STAI-Trait	57.5	(13.2)	60.0	(12.9)	61.8	(11.7)	0.543	0.585	NS
QOL	3.1	(0.5)	2.9	(0.5)	2.8	(0.5)	1.671	0.201	NS
GAF	58.3	(10.4)	55.0	(11.8)	49.5	(8.5)	3.690	0.033	ER > NR *

**Note.** Continuous variables are presented as mean and standard deviation (SD), and categorical variables as number (n) and percentage (%). Continuous variables were compared using one-way analysis of variance, followed by Bonferroni-adjusted post hoc comparisons when the overall test was significant. Categorical variables were compared using Fisher’s exact test; when the overall test was significant, pairwise Fisher’s exact tests with Bonferroni correction were performed. NS indicates that no pairwise comparison remained statistically significant after Bonferroni correction. * *p* < 0.05; ** *p* < 0.01. **Abbreviations:** ER, early remission; DR, delayed remission; NR, non-remission; SSRI, selective serotonin reuptake inhibitor; Y-BOCS, Yale–Brown Obsessive–Compulsive Scale; SDS, Self-Rating Depression Scale; STAI, State–Trait Anxiety Inventory; QOL, quality of life; GAF, Global Assessment of Functioning.

**Table 3:** Treatment Characteristics and Clinical Outcomes at 1 and 2 Years by Remission Trajectory.

	Early Remission		Delayed Remission		Non Remission				
Variable	Mean (SD)/n (%)		Mean (SD)/n (%)		Mean (SD)/n (%)		F/H	*p*	Post Hoc
Treatment characteristics									
Annual number of visits	12.6	(3.4)	12.2	(1.6)	13.1	(3.4)	0.301	0.742	NS
Time to ERP initiation, weeks	11.1	(6.3)	10.2	(3.4)	11.1	(7.0)	0.082	0.921	NS
Visits before ERP initiation, n	4.8	(1.9)	5.7	(1.3)	5.2	(1.0)	1.141	0.329	NS
1-year outcomes									
SSRI use	15.0	(83.3)	9.0	(90.0)	17.0	(89.5)	—	0.862	NS
SSRI dose (imipramine equivalents)	102.8	(69.6)	183.8	(83.8)	173.0	(83.1)	10.478	0.005	NR > ER *; DR > ER *
Antipsychotic use	1.0	(5.6)	3.0	(30.0)	6.0	(31.6)	—	0.110	NS
Antipsychotic dose (chlorpromazine equivalents)	4.2	(17.7)	17.5	(37.4)	39.7	(76.7)	4.196	0.123	NS
Y-BOCS	3.2	(2.6)	12.2	(5.5)	18.4	(6.0)	45.514	<0.001	NR > DR > ER **
SDS	43.6	(11.1)	43.3	(6.0)	50.5	(10.8)	2.647	0.082	NS
STAI-State	44.0	(13.6)	41.4	(9.1)	53.7	(14.5)	3.626	0.035	NS
STAI-Trait	49.6	(13.3)	46.9	(10.0)	57.0	(13.2)	2.493	0.095	NS
QOL	3.3	(0.6)	3.1	(0.5)	3.0	(0.4)	1.254	0.296	NS
GAF	88.3	(12.0)	88.9	(14.5)	74.2	(18.7)	4.723	0.014	ER > NR *
2-year outcomes									
Y-BOCS	3.9	(6.1)	3.4	(1.4)	15.8	(6.5)	26.047	<0.001	NR > ER **; NR > DR **
SDS	44.2	(11.7)	43.6	(8.5)	48.9	(9.2)	1.022	0.37	NS
STAI-State	47.9	(12.5)	43.2	(10.8)	55.1	(11.7)	2.820	0.072	NS
STAI-Trait	55.4	(12.4)	46.6	(13.2)	56.4	(12.2)	1.946	0.157	NS
QOL	3.2	(0.7)	3.4	(0.4)	2.8	(0.4)	2.538	0.093	NS
GAF	91.7	(10.4)	96.7	(5.0)	73.3	(15.0)	13.841	<0.001	ER > NR **; DR > NR **

**Note.** Continuous variables are presented as mean and standard deviation (SD), and categorical variables as number (n) and percentage (%). Normally distributed continuous variables were compared using one-way analysis of variance (ANOVA), followed by Bonferroni-adjusted post hoc comparisons when the overall test was significant. Because medication-equivalent doses showed highly skewed distributions with a substantial number of zero values, these variables were compared using the Kruskal–Wallis test, followed by Bonferroni-adjusted pairwise comparisons when the overall test was significant. Categorical variables were compared using Fisher’s exact test. NS indicates that no pairwise comparison was statistically significant after correction. **Abbreviations:** ER, early remission; DR, delayed remission; NR, non-remission; ERP, exposure and response prevention; SSRI, selective serotonin reuptake inhibitor; Y-BOCS, Yale–Brown Obsessive–Compulsive Scale; SDS, Self-Rating Depression Scale; STAI, State–Trait Anxiety Inventory; QOL, quality of life; GAF, Global Assessment of Functioning. * *p* < 0.05; ** *p* < 0.01.

## Data Availability

The data presented in this study are available from the corresponding author upon reasonable request.
